# Alveolar Abscess

**Published:** 1881-01

**Authors:** Eugene S. Talbot

**Affiliations:** Clinical Lecturer on Dental and Oral Surgery at Central Free Dispensary, Rush Medical College


### ARTICLE III.

ALVEOLAR ABSCESS. By EUGENE S. TALBOT, M.D., D.D.S., Clinical Lecturer on Dental and Oral Surgery at Central Free Dispensary, Rush Medical College. Reported by William A. Synon.

The patient before us, John Kelly, twenty-eight years of age, is suffering from a disease which you will be often called upon to treat in your practice, known as alveolar abscess. We shall devote the remainder of the hour to acquainting ourselves with its cause, symptoms and treatment. This lesion is often ill understood, the term gum-boil being supplemented from a want of knowledge in regard to the pathology of the disease. The primary manifestations of an alveolar abscess is at the apex of the root. As it proceeds to the surface it often terminates in what is commonly known as gum-boil. The word alveolar comes from alveolus, meaning a cavity, a socket: the term showing that the lesion is located in the socket of the tooth. When lumbar abscess, or pelvic abscess, is mentioned, we at once comprehend the location of the lesion. A swelling upon the face or neck, with all the symptoms appertaining to an alveolar abscess, does not always indicate the presence of one. The seat of the disease must be within the alveolar process. We know that the soft tissues of the body are made up of the ultimate elements, carbon, hydrogen, oxygen, nitrogen, sulphur phosphorus, etc., and that when the particles die *en masse*, nature allows the substance to escape in the form of gases. The pulp of a tooth undergoes this same change; death is caused by caries, a blow upon the tooth, constitutional disorders, or from thermal changes. The gases, chiefly $H_2$ S., being unable to pass into the mouth, escape through the apex of the root into the soft tissues. Please bear this point in mind. An alveolar abscess is always pathognomonic of a dead pulp in some one of the teeth. The periosteum, a vascular membrane surrounding the root and lining the alveolar process, affords nourishment to the teeth. This membrane becomes inflamed from the irritation and thickens. Being enclosed between bony walls, the tissues are unable to expand and the tooth is forced from its socket. The patient experiences considerable pain, which ceases upon the slightest pressure, returning again with renewed energy when the force is renewed. These things continuing, plastic lymph is thrown out at the apex of the root and a sac is formed. The surrounding parts becoming involved, the face begins to swell, and the patient suffers the worst form of odontalgia. The sac enlarges and for want of room the pressure causes absorption of the alveolus, and the pus finds its way to the surface. It may point directly here, or it may dissect its way between the bone and periosteum, or between muscles to some remote part, and there form a pouch or pocket. This depends to a great extent upon the tooth and the application used in treatment. The pain ceases as soon as the pus has reached the surface and pressure is removed.

The first symptom observed is a slight soreness about the tooth affected. This increases, followed by a throbbing pain at every beat of the heart. As the pain increases, the tooth elongates, preventing the closing of the jaws. The patient is feverish and tongue coated, bowels constipated, and in a few hours the intermediate tissues distend, the face presenting an appearance scarcely recognizable. His suffering, meanwhile, has become intense, the nervous system has lost its tone and he is completely exhausted. The time varies from two days to two weeks in which the pus forces its way through the alveolus into the soft tissues; the pain ceases then and the tooth returns to its socket. Pus often presses between muscles and lodges in some remote part; this is more noticeable when the seat of the lesion is in the wisdom teeth or incisors. If a wisdom tooth becomes the seat of the trouble, the surrounding parts swell, involving the tonsils and thus interfering with swallowing. The jaws are closed and food is taken with difficulty. Pus sometimes finds an outlet in the larynx, or it makes its way around the neck of the tooth. Frequently it diffuses between the deep and superficial fascia and lodges near the larynx, trachea and clavicle. If the disease is connected with the incisor, the pus often works between the bone and periosteum, forming a tumor in the roof of the mouth. A fistula may form at this locality, or from pressure may cause absorption of the palate bone and discharge into the nares. If the inferior incisor becomes involved, the pus, in obeyance to the law of gravitation, makes its exit through the integument beneath the chin. If the cause is not removed, the abscess becomes chronic and a permanent opening with red granulations about its margins is the result.

If a patient presents himself with a swelling or fistula upon the face or neck, you at once suspect that the trouble proceeds from the teeth. My experience teaches that the teeth are the seat of the majority of these pathological conditions. Make a thorough examination of the jaws to ascertain if there be any roots or decayed teeth. The locality of the swelling will aid you in this direction. The patient may be able to locate the tooth, but the practitioner must not be influenced against his judgment, as the patient may be mistaken owing to the extended inflammation. This might result in extraction of the wrong tooth—a too common occurrence. Satisfy yourself before sacrificing a tooth, and your judgment will be appreciated. In making a superficial examination only, we may not always be successful in locating the particular tooth which causes the trouble. You should all be provided with an oral surgeon's mouth-mirror and a steel instrument similar to a dental excavator. The mouth-mirror is a very convenient instrument for reflecting the light not only in every part of the oral cavity, but in the larynx as well. The steel instrument is an important aid; with it tap the teeth upon their grinding surfaces, commencing with the posterior tooth and advancing forward. When the particular tooth is reached, the patient will respond to the pain occasioned by the blow. In tapping the molars, it is essential that the whole surface should be gone over, because the seat of the disease may be located at the apex of any one of the roots, and unless the blow is directed over the affected root, the patient will not respond. Sometimes this proceeding is of little value. This is the case when the abscess has become chronic and fistulæ are established and the soreness of the tooth has passed away. Place the patient in a position that with the head thrown back a strong light may pentrate the oral cavity. With a mouth-mirror reflect the light upon the teeth and observe the difference in color. When death of the pulp occurs, the tooth generally presents a bluish appearance. There are two reasons for this: one, that the animal matter is drawn into the tubule of the dentine by capillary attraction; the other, that the $H_2$ S unites with the iron in the blood, forming sulphide of iron. This discoloration is not always noticeable, particularly in older people; as, when age advances, the tubuli become smaller, so these substances are prevented from entering. If these attempts should fail to accomplish the diagnosis, another excellent plan is to warm the instrument used in tapping the teeth in a spirit lamp, and apply it to the crowns of the teeth. When it touches the teeth in which the pulp is alive, the patient will immediately respond, when the ones in which a dead pulp is located will be void of sensation. Hot and cold water may also be applied with a syringe—the cold having no effect, the hot producing pain.

Abscesses connected with the wisdom teeth of the lower jaw manifest themselves by a similar but a more severe line of symptoms than when connected with other teeth. The swelling takes place in the parotid region, the muscles of mastication become rigid, jaws set, the patient unable to swallow. The face is swollen to an enormous size and the pain is intense. Abscesses caused by the eruption of these teeth and those caused by the death of their pulps must not be confounded. Those caused from the eruption of the wisdom teeth are not alveolar abscesses. They differ in their locations, being situated around the crown, instead of at the apex of the root. They are caused by pressure upon the surrounding parts, in nature's efforts to force the teeth into place, and require a different course of treatment. Swellings and discharges in the roof of the mouth and nares are often a result of abscesses at the apex of the incisors. They occur more frequently when connected with the laterals. The pus dissects its way between the bone and periosteum, forming a pocket in the roof of the mouth, where it discharges into the oral cavity, or causes, by pressure, absorption of the maxillary bones discharging its contents into the nares. In some cases, abscesses have discharged their contents as far back as the soft palate, leading the practitioner to suspect caries of the bone. The antrum, the base of which extends from the canine tooth back, including the wisdom tooth, sometimes becomes involved, discharging its contents into the middle meatus of the nose, or, being encysted, produces a bulging of the antral walls. Fistulæ upon the face, the result of alveolar abscess, are often mistaken for necrosis, caries and cancer. Two cases have come to my notice in the past two years; the first was a young lady who had been treated by different physicians more than four years for cancer beneath the infra-orbital foramin. While there was no pain or constitutional disturbance, the patient had become quite anæmic, and the expression of countenance indicated intense mental anxiety. Finally, she drifted into the hands of a physician who suspected the cause and called to his assistance a skillful dentist. They diagnosed the lesion, the seat of which was located at the apex of the canine tooth. This was treated, the abscess cured and the patient recovered. The next case was a woman with a fistula at the lower border of the chin, caused by an abscess at the root of the inferior incisor. She submitted to a surgical operation, and to the surprise of the operator no dead bone was found, the removal of the tooth relieving the trouble. It is not always easy to make out a diagnosis when caries and necrosis of the jaws are characterized by a similar line of symptoms. A plan which I believe to be safe and sure is by exclusion, as I have already stated. In these cases, suspect the teeth to be the seat of the difficulty.

Pass your probe into the sinus, notice the direction it takes before meeting an obstruction; should it prove to be a hard, smooth circumscribed mass, it (together with the direction which the probe takes) will aid you in extending your search in the cavity of the mouth and limit the extent of field to be explored. With the aid of the instrument, mouth-mirror, and heat you will be able to locate the tooth. Abscesses are as likely to arise from filled teeth as from roots, their pulps dying from thermal changes taking place through the fillings. If you are confident that the teeth do not cause the trouble, suspect dead or carious bone. You are familiar with the peculiar, soft, spongy honey-comb nature of the tissue and the readiness with which the probe penetrates. We do not find in alveolar abscess that dense inflammatory deposit extended in size often with more than one sinus and pus offensive which are found in necrosis. Having ascertained that neither the teeth or bone cause the trouble, we shall next suspect a cancerous condition. In treating alveolar abscess and its complications, the simplest, surest and quickest means of eradicating it would be to remove the offending organ, but in these enlightened times in the hands of a specialist very few of the organs are sacrificed. The loss of a tooth impairs the whole dentine. Therefore efforts should invariably be made to preserve them. Should it be deemed best to extract the tooth, the following treatment will prove simple and efficacious: an injection of tr. iodine or carbolic acid into the sinus; if pus cells have commenced to form, we apply hot figs or raisins to the gum directly over the rebellious root. Also an excellent and more convenient remedy is one made from the following prescription:

Tr. capsicum,<br>Alcohol,<br>Chloroform, } of each ℥j applied on a bit of cotton to the gum as before, pointing the pus into the mouth. Hot applications must never be made on the face, as it would point the pus to the surface; hypodermic injections of morphia may be given to relieve the pain. In lancing an abscess, pass the bistoury into the mouth, keeping the back of the blade against the jaw as a guide to avoid the facial artery. Never lance an abscess upon the face; an ugly scar would be the result, and should be considered malpractice. In abscesses resulting from death of the pulp by fillings, we should remove the fillings to allow the gases to escape, after which the root can be treated and the tooth refilled. Should you be fortunate enough to see the patient when the first symptoms manifest themselves, endeavor to avoid more serious results by freely lancing the gums, carrying the knife through the process to reach the inflamed periosteum and apply trs. aconite and iodine to the parts. Should this fail, then force the knife, or drill through the alveolar process at the apex of the root, and in this way give vent to the inflamed product, administering saline cathartics. We find the eruption of the wisdom teeth particularly difficult to treat, requiring the use of the lancet often to remove the mucous membrane. Frequently the space between the second molar and the angle of the jaw is insufficient to allow the wisdom tooth to come up into place. When possible, we consider it wisest to remove the wisdom tooth; but if the tooth has not advanced enough to extract it, we remove the second molar rather than undergo any extended operation. When the antrum is involved, it may be necessary to remove the tooth causing the trouble. As the roots often extend into this cavity, it is quite difficult to treat an abscess in this locality. After the root is removed, if the secretion should not flow out, pass a drill or trephine up into the socket through the opening made by the root. Having removed the cause, cleanse the cavity with warm water, this being all required.

